# Manganese superoxide dismutase deficiency exacerbates the mitochondrial ROS production and oxidative damage in Chagas disease

**DOI:** 10.1371/journal.pntd.0006687

**Published:** 2018-07-25

**Authors:** Jake J. Wen, Nisha Jain Garg

**Affiliations:** 1 Department of Microbiology and Immunology, University of Texas Medical Branch (UTMB), Galveston, Texas, United States of America; 2 Department of Pathology, UTMB, Galveston, Texas, United States of America; 3 Institute for Human Infections and Immunity, UTMB, Galveston, Texas, United States of America; Albert Einstein College of Medicine, UNITED STATES

## Abstract

In this study, we have investigated the effects of manganese superoxide dismutase (SOD2 or MnSOD) deficiency on mitochondrial function and oxidative stress during Chagas disease. For this, C57BL/6 wild type (WT) and MnSOD^+/-^ mice were infected with *Trypanosoma cruzi* (*Tc*), and evaluated at 150 days’ post-infection that corresponded to chronic disease phase. Genetic deletion of SOD2 decreased the expression and activity of MnSOD, but it had no effect on the expression of other members of the SOD family. The myocardial expression and activity of MnSOD were significantly decreased in chronically infected WT mice, and it was further worsened in MnSOD^+/-^ mice. Chronic *T*. *cruzi* infection led to a decline in mitochondrial complex I and complex II driven, ADP-coupled respiration and ATP synthesis in the myocardium of WT mice. The baseline oxidative phosphorylation (OXPHOS) capacity in MnSOD^+/-^ mice was decreased, and it had an additive effect on mitochondrial dysregulation of ATP synthesis capacity in chagasic myocardium. Further, MnSOD deficiency exacerbated the mitochondrial rate of reactive oxygen species (ROS) production and myocardial oxidative stress (H_2_O_2_, protein carbonyls, malondialdehyde, and 4-hydroxynonenal) in Chagas disease. Peripheral and myocardial parasite burden and inflammatory response (myeloperoxidase, IL-6, lactate dehydrogenase, inflammatory infiltrate) were increased in all chagasic WT and MnSOD^+/-^ mice. We conclude that MnSOD deficiency exacerbates the loss in mitochondrial function and OXPHOS capacity and enhances the myocardial oxidative damage in chagasic cardiomyopathy. Mitochondria targeted, small molecule mitigators of MnSOD deficiency will offer potential benefits in averting the mitochondrial dysfunction and chronic oxidative stress in Chagas disease.

## Author summary

Infection by *Trypanosoma cruzi* parasitic protozoan remains endemic in Latin America. After acute parasitemia phase is controlled by host immune system, infected individuals remain clinically silent but manifest a number of micro and macro cardiac injuries for several years. Eventually many of the infected individuals develop chronic cardiomyopathy that leads to heart failure and sudden death. Cardiac muscle cells are rich in mitochondria and manganese superoxide dismutase (MnSOD) is the chief superoxide scavenging enzyme in the mitochondria. In this study, we show that a deficiency of MnSOD exacerbates the *T*. *cruzi* induced mitochondrial dysfunction of the electron transport chain and energy production in the heart. Further, MnSOD deficiency resulted in increased mitochondrial release of oxidants and caused excessive oxidative damage in the chagasic heart. Our results suggest that small molecule agonists of MnSOD will have potential utility as adjuvant therapy in preventing the development of chronic Chagas disease in infected individuals.

## Introduction

Chagasic cardiomyopathy is caused by the protozoan *Trypanosoma cruzi* (*Tc* or *T*. *cruzi*) [1]. Infected individuals exhibit an acute phase of peak blood parasitemia that is resolved in 2-3-months. Approximately, 30–40% of infected individuals progress to present ventricular fibrillation, thromboembolism, and congestive heart failure [2,3].

During the fetal heart development, cardiomyocytes are dependent on glycolysis as a source of energy. In the mature heart, fatty acid oxidation coupled with oxidative metabolism in mitochondria provides >90% of the energy required for continual contraction to supply the body with blood [4]. The reduced substrates (NADH, FADH2) deliver electrons from complex I (CI) and complex II (CII) of the electron transport chain through complex III (CIII) and complex IV (CIV) to oxygen, causing protons to be pumped across the mitochondrial inner membrane, and setting proton motive force that drives protons back through the ATP synthase (complex V), forming ATP from ADP and phosphate [5]. During this process, electrons may leak from the respiratory chain and react with oxygen to form superoxide [6]. The Q1 semi-ubiquinone of complex III in the electron transport chain is believed to be the major site of superoxide production. Superoxide anions are charged molecules that directly or indirectly contribute to formation of other reactive oxygen species (ROS), and can result in cellular oxidative damage.

Superoxide dismutases (SODs) are metallo-enzymes that catalyze the conversion of superoxide anion (O_2_•-) to hydrogen peroxide (H_2_O_2_). In higher eukaryotes, SODs are expressed by different genes, and have historically been designated as copper- and zinc-containing, cytosolic, homo-dimer enzyme (CuZnSOD or SOD1), manganese-containing, mitochondrial, homo-tetramer enzyme (MnSOD or SOD2), and copper- and zinc-containing tetramer enzyme that is secreted to extracellular spaces (ECSOD or SOD3) [7]. The SOD2 enzyme binds one manganese ion per subunit, and is the major mitochondrial antioxidant.

We have previously documented an increase in mitochondrial reactive oxygen species (mtROS) in chagasic hearts [8,9]. In this study, we aimed to determine if MnSOD deficiency worsens the mitochondrial health during *Trypanosoma cruzi* infection and Chagas disease. For this, C57BL/6 WT and MnSOD^+/-^ mice were infected with *T*. *cruzi*, and we examined the effect of MnSOD deficiency on mitochondrial function, and oxidative and inflammatory stress in chagasic heart. We discuss the benefits of mitochondria targeted, small molecule mitigators of MnSOD deficiency in offering potential therapy against mitochondrial dysfunction and chronic oxidative stress in Chagas disease.

## Materials and methods

### Ethics statement

All animal experiments were performed according to the National Institutes of Health Guide for Care and Use of Experimental Animals, and approved by the Institutional Animal Care and Use Committee (IACUC) at the University of Texas Medical Branch, Galveston (protocol number: 0805029).

### Mice, parasites, and cell culture

C57BL/6 wild-type (WT) mice were purchased from Harlan Laboratories (Indianapolis, IN). MnSOD^+/-^ mice (C57BL/6 background) were kindly provided by Dr. H. Van Rammen, and have been described previously [10,11] [12]. All mice were bred at the UTMB animal facility. *T*. *cruzi* trypomastigotes (SylvioX10/4) were propagated by *in vitro* passage in C2C12 immortalized mouse myoblast cells. Mice (5-6-weeks-old, body weight: 18.23 ± 1.67 g) were infected with *T*. *cruzi* (10,000 trypomastigotes/mouse, intraperitoneal), and sacrificed at 150 days’ post-infection (pi) corresponding to chronic disease phase [13]. Sera/plasma and tissue samples were stored at 4°C and -80°C, respectively. Protein levels in all samples were determined by using the Bradford Protein Assay (Bio-Rad, Hercules CA). All chemicals were of molecular grade (>99% pure) and purchased from Sigma-Aldrich (St. Louis, MO).

### Genotyping and tissue parasite burden

Tail biopsies from young pups or heart tissue sections (10 mg) from chronically infected WT and MnSOD^+/-^ mice were subjected to Proteinase-K lysis and total DNA was extracted and purified by phenol/chloroform extraction/ethanol precipitation method [14]. Tail DNA samples were analyzed by traditional PCR for genotyping WT and MnSOD^+/-^ mice [15].

To examine, blood and tissue parasite burden, total DNA (100 ng) was used as template with *Tc*18SrDNA-specific primers. A real-time quantitative PCR reaction was performed for 35 cycles with SYBR Green Supermix (Bio-Rad) on an iCycler thermal cycler. Cycling parameters were as follows: Initial denaturation at 95°C for 5 min, denature at 95°C for 15 sec, anneal at 60°C for 30 sec, then denature-anneal cycling for 34 more times. Single product amplification was confirmed in the melt curve analysis. The threshold cycle (*C*_*T*_) values for target DNAs were normalized to the *C*_*T*_ values for the GAPDH housekeeping gene sequence (ΔC_*T*_), and relative parasite burden was calculated as 2^-ΔCt^ (ΔC_t_ = Ct_*Tc*18SrDNA_—Ct_GAPDH_) [16]. All oligonucleotides are listed in S1 Table.

### Reverse transcription, quantitative PCR (RT-qPCR)

Heart tissue sections (10 mg) were homogenized in TRIzol reagent (Invitrogen, Carlsbad, CA; weight/volume ratio, 1:10). Total RNA was extracted, precipitated, and purified of contaminating DNA, and analyzed for quality (OD_**260/280**_ ratio ≥ 2.0) and quantity (OD_260_ of 1 = 40 μg/ml RNA). Purified RNA (2 μg) was reverse transcribed by using an iScript kit (Bio-Rad), and cDNA was used as template with gene-specific oligonucleotide pairs (S1 Table) in real-time, quantitative PCR reaction for 35 cycles, as described above. The Ct values for target mRNA were normalized to geometric mean of GAPDH mRNA, and fold change in gene expression was calculated as 2^−ΔΔCt^, where ΔC_t_ represents the C_t_ (sample)—C_t_ (control) [17].

### Tissue homogenates and mitochondrial fractions

Tissue sections (tissue: buffer ratio, 1:10 w/v) were homogenized in RIPA buffer (Cell Signaling, Danvers, MA, #9806,), centrifuged at 10,000 g, and supernatants were used as tissue lysates. To isolate mitochondria, freshly harvested heart tissues were suspended in homogenization buffer (50 mM Tris-HCl, pH 7.4, 5 mM MgCl_2_, 1 mM DTT, 25 μg/ml spermine, 25 μg/ml spermidine, and protease inhibitor cocktail) containing 250 mM sucrose; tissue: buffer ratio, 1:20) and homogenized at 4°C by using a dounce homogenizer. The homogenates were centrifuged at 800 g for 15 min at 4°C, and supernatants were then centrifuged at 6000 g for 15 min. The resultant pellets were stored as mitochondrial fractions [18]. Western blotting was performed to examine the nuclear (Lamin B), and mitochondrial (COIV subunit) proteins in all samples. Mitochondrial fractions that exhibited > 6% of contaminants were re-centrifuged as described above to ensure purity [19].

### Western blotting

Heart homogenates (30 μg protein) were electrophoresed on a 4–15% Mini-Protein TGX gel, and proteins were wet-transferred to a PVDF membrane. Membranes were blocked with 5% non-fat dry milk (NFDM) in 50 mM Tris-HCl (pH 7.5) / 150 mM NaCl (TBS), washed with TBS-0.1% Tween 20 (TBST) and TBS, and incubated overnight at 4°C with antibodies (1: 1000 dilution) against MnSOD (Abcam, Cambridge, UK, Ab13533) or GAPDH (Cell Signaling, clone 14C10). Membranes were washed, incubated with HRP-conjugated secondary antibody (1:5000 dilution, Southern Biotech, Birmingham, AL), and Amersham ECL Plus system (GE Healthcare, Pittsburgh, MA) was used to develop the signal. An ImageQuant LAS4000 system (GE Healthcare) was used to visualize and digitize the images, and a Fluorchem HD2 Imaging System (Alpha-Innotech, San Leandro, CA) was used to perform densitometry analysis of the images [19].

### MnSOD activity

MnSOD activity in tissue homogenates was measured by using a Superoxide Dismutase Assay kit (Cayman Chemicals, Ann Arbor, MI, #706002). One unit of SOD was defined as the amount of MnSOD needed to exhibit 50% neutralization of superoxide radical (standard curve: 0.005–0.05 U/ml recombinant CuZnSOD).

### Mitochondrial respiration and ROS production

Mitochondrial respiration was measured by using a Mitocell S200A Respirometry System (Strathkelvin, Motherwell, UK) [20]. Briefly, freshly isolated mitochondria (200 μg) were added to the mitocell in 0.5 ml of MSP buffer (225 mM mannitol, 75 mM sucrose, 20 mM KH_2_PO4/K_2_HPO4 pH 7.6). After equilibration, electron flow was supported by 10 mM pyruvate / 10 mM glutamate / 2.5 mM malate (P+G+M, complex I substrates), and CI-dependent state 4 respiration was recorded. Then 230 μM ADP was added, and ADP-coupled, CI-dependent state 3 respiration was recorded. Next, 6.25 μM rotenone was added to inhibit electron flow from complex I, mitochondria were energized with 10 mM succinate (complex II substrate), and C-II-dependent state 3 respiration was recorded. Finally, 1 μM antimycin (inhibits electron flow at complex III) and CII-supported state 4 was recorded. A high respiratory control ratio (RCR = state 3/state 4) suggests mitochondrial capacity for substrate oxidation and ATP turnover and a low proton leak. The ADP/O ratio (mitochondrial ATP production capacity) was calculated as decrease in O_2_ concentration during state 3 respiration per O atom consumed.

To measure mitochondrial rate of ROS generation, isolated mitochondria (25-μg protein) were added in triplicate to 96-well, black flat-bottomed plates in 100 μl of reaction buffer (10 mM Tris-HCl at pH 7.4, 250 mM sucrose, 1 mM EDTA), and energized with complex I or complex II substrates, as above. Mitochondria were loaded with 30-μM dihydroethidium (DHE), and formation of fluorescent ethidium by intra-mitochondrial ROS was recorded at Ex_498nm_/Em_598nm_, by using a SpectraMax M2 microplate reader (Molecular Devices, San Jose, CA). As an alternate method, mitochondria were incubated with 33-μM amplex red and 0.1 U/ml of horseradish peroxidase (HRP), and HRP-catalyzed, ROS-mediated oxidation of amplex red to fluorescent resorufin was recorded at Ex_563nm_/Em_587nm_. Standard curves were prepared with etihidium (1–15 μM) and H_2_O_2_ (50 nM–5 μM).

### Oxidant levels

To measure the H_2_O_2_ levels, 50 μl of tissue lysates (100 μg) were added in triplicate to flat-bottom (dark-walled) 96-well plates. Then 100 μl of reaction mixture containing 0.05 M sodium phosphate, pH 7.4, 33 μM amplex Red, and 0.1 U/ml HRP was added. The plates were incubated for 30 min in dark, and ROS levels were recorded as above.

Protein carbonyls in tissue homogenates were measured by a colorimetric protein carbonyl assay (Cayman Chemicals, #10005020).

Malonyldialdehyde (MDA) levels were measured by a TBARS assay (Cayman Chemicals, #10009055). Concentration of lipid peroxides was calculated as an MDA equivalent using the extinction coefficient for the MDA–TBA complex of 1.56x10^5^ M^−1^ cm^−1^ at 532 nm.

To examine the 4-hydroxynonenal (4-HNE) levels, tissue lysates were subjected to Western blotting with anti-4-HNE antibody (Abcam, ab46545, 1:1000 dilution).

### Inflammatory markers

The interleukin 6 (IL-6) cytokine levels in plasma samples were measured by using IL-6 sandwich ELISA kit (BD Biosciences, San Jose, CA). The change in absorbance as a measure of cytokine concentration was monitored at 450 nm by using a SpectraMax M5 spectrophotometer (Molecular Devices). A standard curve was prepared with 0–1,000 pg/ml of recombinant cytokine.

To measure myeloperoxidase (MPO) levels, plasma samples (10 **μ**g of protein) were added in triplicate to 0.53-mmol/L o-dianisidine dihydrochloride and 0.15 mmol/L H_2_O_2_ in 50 mmol/L potassium phosphate buffer (pH, 6.0). Reaction was stopped after 5 minutes, and absorbance was measured at 460 nm on a SpectraMax 190 microplate reader. One unit of MPO was defined as that degrading 1 nmol H_2_O_2_/min (ε = 11 300M^−1^.cm^−1^).

### Histology

Heart tissue sections were fixed in 10% buffered formalin, dehydrated in absolute ethanol, cleared in xylene, and embedded in paraffin. Five-micron tissue sections were subjected to staining with hematoxylin and eosin (H&E) o at the Research Histopathology Core at the UTMB, and evaluated by light microscopy using an Olympus BX-15 microscope (Center Valley, PA) equipped with a digital camera and Simple PCI software (v.6.0; Compix, Sewickley, PA). Myocarditis (presence of inflammatory cells) in H&E stained tissue sections was scored as 0 (absent), 1 (focal/mild, ≤1 foci), 2 (moderate, ≥2 inflammatory foci), 3 (extensive coalescing of inflammatory foci or disseminated inflammation), and 4 (diffused inflammation, tissue necrosis, interstitial edema, and loss of integrity). Inflammatory infiltrates was characterized as diffused or focal depending upon how closely the inflammatory cells were associated [21].

### Data analysis

The WT and MnSOD^+/-^ mice were randomly assigned to *Tc* infection and no infection groups (n = 5 mice per group per experiment). All experiments were conducted at least twice, and a minimum of duplicate observations were acquired for each sample. All data were analyzed by using a GraphPad Prism 5 software (La Jolla, CA) and expressed as mean ± standard error mean (SEM). Statistical significance was calculated by the student’s t test (for comparison of 2 groups) and one-way analysis of variance (ANOVA) with Tukey's post hoc test (for comparison of more than two groups). Significance is presented by ^a^ MnSOD^+/-^ vs. WT, ^b^ WT.*Tc* vs. WT, ^c^ MnSOD^+/-^.*Tc* vs. MnSOD^+/-^, ^d^ MnSOD^+/-^.*Tc* vs. WT.*Tc*, *and* ^e^ MnSOD^+/-^.*Tc* vs. WT mice (p value < 0.05).

## Results

**Fig 1:**
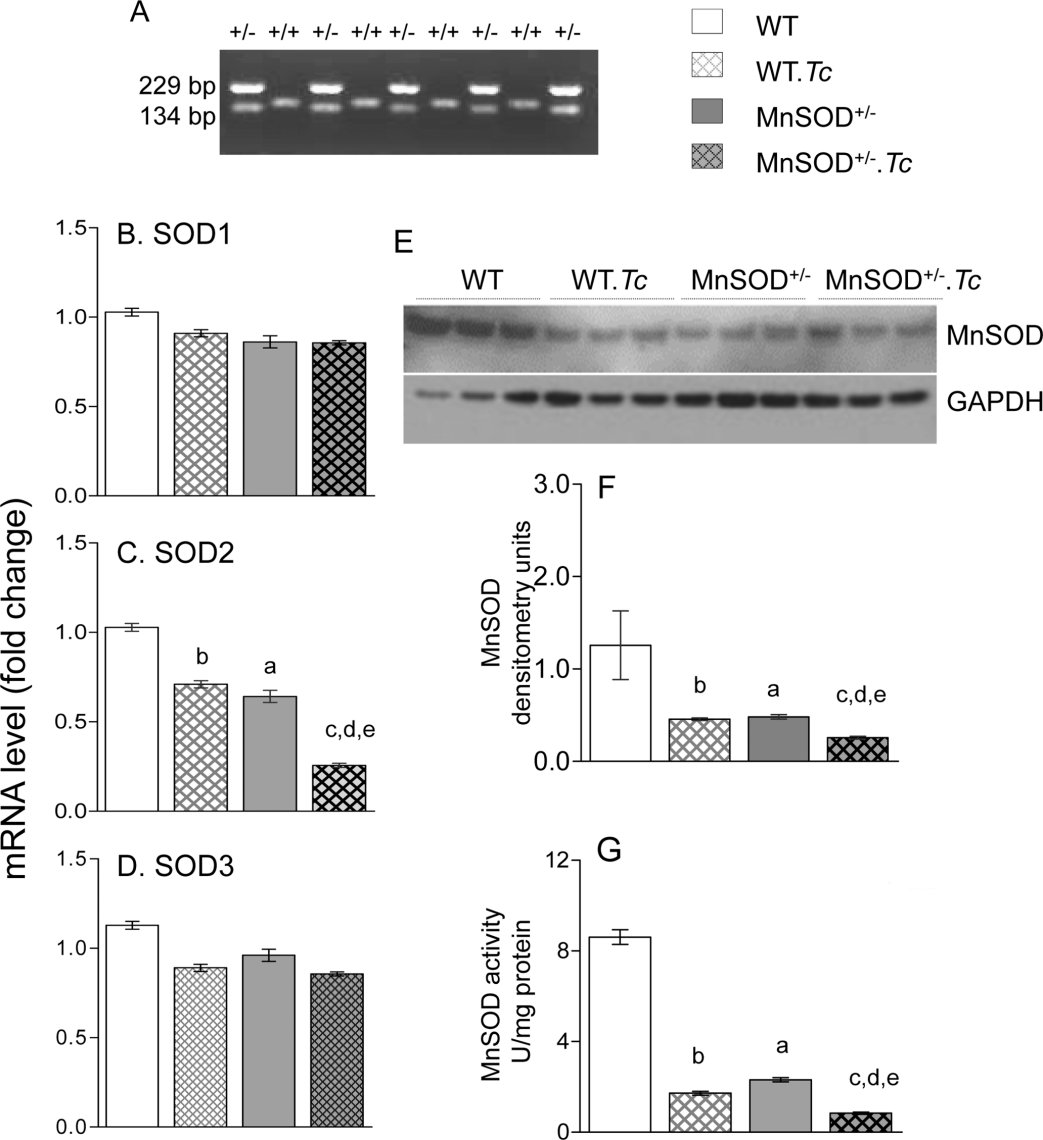
Expression and activity of superoxide dismutases in chagasic mice. C57BL/6 (WT and MnSOD^+/-^) mice were infected with *Trypanosoma cruzi* (10,000 *Tc*/mouse), and sacrificed at 150 days’ post-infection (pi) corresponding to chronic disease phase. **(A)** Total DNA was used as template for genotyping the MnSOD^+/-^ (vs. WT) mice. The amplification of a 229 bp band indicates the integration of xx within *MnSOD* gene **(B-D)** Reverse transcription—quantitative PCR (RT-qPCR) evaluation of myocardial levels of *SOD1* ***(B)***, *SOD2* ***(C)*** *and SOD3* ***(D)*** mRNAs was performed in WT and MnSOD^+/-^ mice before infection and at 150 days pi. The data were normalized to *GAPDH* mRNA (n = 6 mice per group, triplicate observations per mouse). **(E&F)** Representative Western blot images for myocardial levels of MnSOD and GAPDH (loading control) are shown (***E***, n = 3 mice per group). Densitometry analysis was performed for Western blot gels representing n = 6 mice per group (duplicate observations per mouse), and data were normalized to GAPDH levels ***(F)***. **(G)** Heart homogenates were subjected to differential centrifugation to isolate mitochondria. Mitochondrial MnSOD activity was determined by a spectrophotometric assay (n = 6 mice per group, duplicate observations per mouse). Data in bar graphs are plotted as mean value ± SEM. Statistical significance are marked as ^a^MnSOD^+/-^ vs. WT, ^b^WT.*Tc* vs. WT, ^c^MnSOD^+/-^.*Tc* vs. MnSOD^+/-^, ^d^MnSOD^+/-^.*Tc* vs. WT.*Tc*, and ^e^MnSOD^+/-^.*Tc* vs. WT (p value < 0.05).

The MnSOD^+/-^ and WT mice were genotyped to confirm the presence of one and two copies of the *MnSOD* gene, respectively (Fig 1A). We then evaluated the effect of *Tc* infection on the expression levels of the mammalian superoxide dismutases, including copper- and zinc-containing, cytosolic, homodimer enzyme (SOD1 or CuZnSOD), manganese-containing, mitochondrial, tetramer enzyme (SOD2 or MnSOD), and copper- and zinc-containing tetramer enzyme that is secreted to extracellular spaces (SOD3 or ECSOD) in WT and MnSOD^+/-^ mice. The myocardial levels of *SOD1* and *SOD3* mRNAs were not statistically different in WT and MnSOD^+/-^ mice, and *Tc* infection had no effects on *SOD1* and *SOD3* expression in WT and MnSOD^+/-^ mice (Fig 1B & 1D). The basal levels of MnSOD mRNA, protein, and enzymatic activity were decreased by 37.6%, 61.6%, and 73%, respectively, in the myocardium of MnSOD^+/-^ (vs. WT) mice (Fig 1C and 1E–1G, ^a^p<0.05). The myocardial levels of MnSOD mRNA, protein and activity in chronically infected WT.*Tc* (vs. WT) mice were decreased by 31%, 63% and 80%, respectively (Fig 1C and 1E–1G ^b^p<0.05). The *T*c-induced MnSOD deficiency was further worsened in MnSOD^+/-^ mice as was evidenced by 63%, 56%, and 51% decline in MnSOD mRNA, protein, and activity, respectively, in MnSOD^+/-^.*Tc* (vs. WT.*Tc*) mice (Fig 1C and 1E–1G, ^d^p<0.05) mice. Together, these results suggest that a) genetic deletion of *SOD2* decreased the expression and activity of MnSOD, but it had no effect on the expression of other members of the SOD family, b) myocardial expression of *SOD1* and *SOD3* were not changed in response to *Tc* infection, c) the expression and activity of MnSOD was significantly decreased by chronic *Tc* infection in WT mice, and it was further worsened in MnSOD^+/-^ mice.

**Fig 2:**
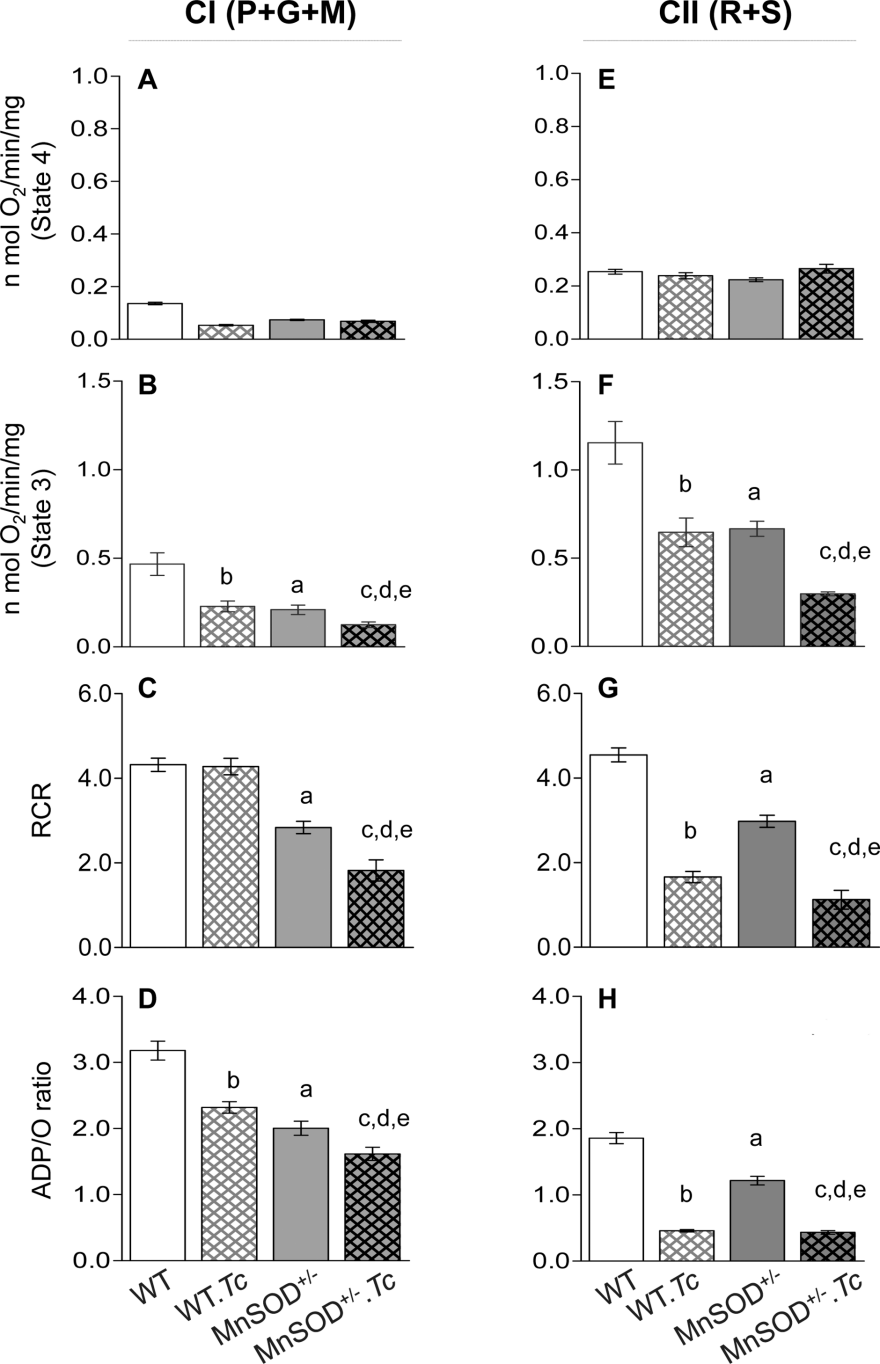
Effects of MnSOD deficiency on respiratory properties of cardiac mitochondria in chagasic mice. C57BL/6 (WT and MnSOD^+/-^) mice were sacrificed in chronic disease phase and cardiac mitochondria were isolated as described in Materials and Methods. Freshly isolated mitochondria were incubated with pyruvate/glutamate/malate (P+G+M) to support electron flow through complex I **(A-D)** or with rotenone + succinate (R+S) to support electron flow through complex II (**E-H**). The mitochondrial respiratory function was recorded by using a S200A respirometry system. Bar graphs show the substrate supported state 4 respiration ***(A&E)***, ADP-coupled state 3 respiration ***(B&F)***, respiratory control ratio (RCR = state 3/state 4, ***C&G***), and ADP/O ratio **(*D&H*)** that measures the amount of ADP phosphorylated per O atom consumed. Data are plotted as mean value ± SEM (n ≥ 6 mice per group, triplicate observations per mouse). Significance is presented by a-e as described in Fig 1 (p value < 0.05).

We next determined the effects of MnSOD deficiency on mitochondrial health in Chagas disease. There were no discernible differences in mitochondrial yield between WT and MnSOD^+/-^ mice. The effects of MnSOD deficiency on complex I supported mitochondrial respiration (± *Tc*) are shown in Fig 2A–2D. No significant change in state 4 respiration was observed in cardiac mitochondria of WT and MnSOD^+/-^ mice (Fig 2A), while basal level of complex I driven state 3 was decreased by 44–55%, and contributed to ~35% decline in RCR in cardiac mitochondria of MnSOD^+/-^ (vs. WT) mice (Fig 2B & 2C, ^a^p<0.05). The chronically infected WT.*Tc* (vs. WT) mice exhibited a 60.7% and 51% decline in myocardial, complex I driven state 4 and state 3 respirations, respectively (Fig 2A & 2B, ^b^p<0.05), and no change in RCR value (Fig 2C). In MnSOD^+/-^ mice; *T*. *cruzi* infection worsened the complex I driven state 3 respiration by 40% (vs. uninfected/MnSOD^+/-^, Fig 2B, ^c^p<0.05). The complex I-dependent ADP/O ratio (indicates the ATP synthesis rate) was decreased by 37% and 27% respectively, when MnSOD deficiency and chronic *Tc* infection were present individually, and by 49% in MnSOD^+/-^.*Tc* (vs. WT) mice (Fig 2D, ^a-e^p<0.05).

**Fig 3:**
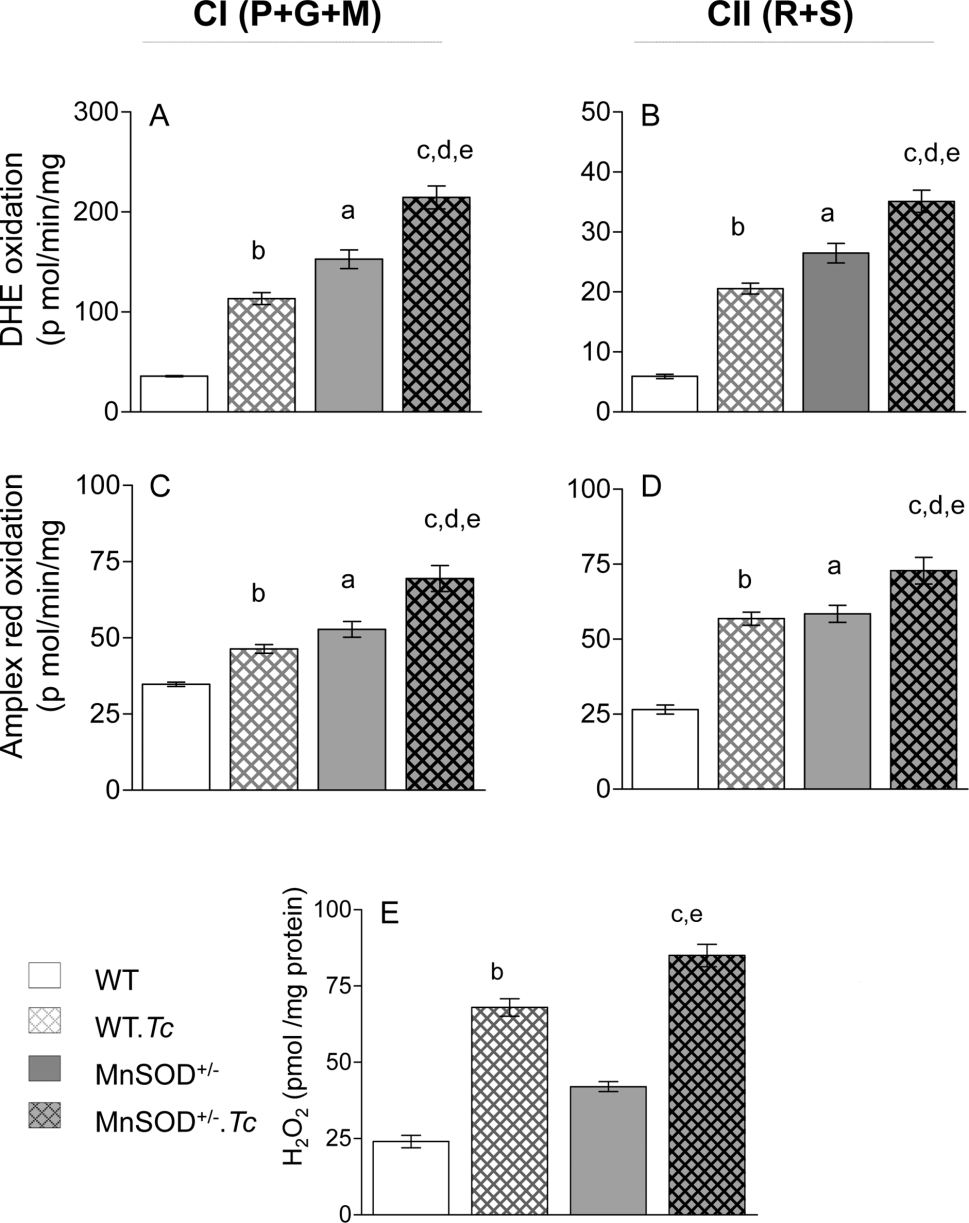
Effects of MnSOD deficiency on mitochondrial and cardiac ROS in chagasic mice. C57BL/6 (WT and MnSOD^+/-^) mice were sacrificed at 150 days pi. **(A-D)** Freshly isolated cardiac mitochondria were incubated in presence of pyruvate/glutamate/malate (P+G+M) to support electron flow through complex I ***(A&C)*** or with rotenone/succinate (R+S) to support electron flow through complex II ***(B&D)***. The ROS production was measured by oxidation of dihydroethidium (DHE) to fluoresecent ethidium ***(A&B)*** and amplex red oxidation to resorufin ***(C&D)***. **(E)** Myocardial H_2_O_2_ levels were determined by an amplex red assay as described in Materials and Methods. Data are plotted as mean value ± SEM (n = 6 mice per group, triplicate observations per mouse). Statistical significance are marked as ^a^MnSOD^+/-^ vs. WT, ^b^WT.*Tc* vs. WT, ^c^MnSOD^+/-^.*Tc* vs. MnSOD^+/-^, ^d^MnSOD^+/-^.*Tc* vs. WT.*Tc*, and ^e^MnSOD^+/-^.*Tc* vs. WT (p value < 0.05).

The complex II driven respiratory parameters in WT and MnSOD^+/-^ mice (± *Tc*) are shown in Fig 2E–2H. As above, complex II supported state 4 was not changed by MnSOD deficiency or *Tc* infection (Fig 2E). However, complex II driven state 3 and RCR were decreased by 43–44% and 34–65% respectively, by MnSOD deficiency (MnSOD^+/-^ vs. WT, ^a^p<0.05) or chronic *Tc* infection (WT.*Tc* vs. WT, ^b^p<0.05), and MnSOD deficiency and *Tc* infection together resulted in >70% decline in complex II driven coupled respiration and RCR in the myocardium (Fig 3F & 3G, compare MnSOD^+/-^.*Tc* vs. WT, ^e^p<0.05). Likewise, complex II supported ADP/O ratio was decreased by 34% due to MnSOD deficiency or chronic infection only (Fig 3H, ^a,b^p<0.05), and by >75% with presence of MnSOD deficiency and chronic infection together (Fig 3H, ^e^<0.05). Together, our finding of no change in state 4 above the basal level suggest that mitochondrial preparations were not damaged or uncoupled, and basal chemiosmosis gradient was not altered by MnSOD deficiency. Yet, MnSOD deficiency and chronic infection independently caused a decline in complex I and complex II supported, ADP-coupled respiration and ATP synthesis in murine myocardium, and the ATP synthesis capacity was increasingly deteriorated in MnSOD^+/-^ mice with chagasic disease.

The MnSOD is an essential mitochondrial antioxidant that detoxifies free radical superoxide (O_2_•-) generated by mitochondrial respiration. We next determined if MnSOD deficiency exacerbates the mtROS production in chagasic myocardium. For this, we incubated the isolated cardiac mitochondria with P+G+M to energize the electron transport chain at complex I, or with rotenone and succinate to inhibit the complex I and energize the electron transport chain at complex II. Then, we measured the rates of DHE oxidation to fluorescent hydroethidium (detects intra-mitochondrial ROS) and of amplex red to resorufin (detects ROS release). The CI- and CII-driven DHE and amplex red oxidation were increased by 330–350% (Fig 3A & 3B) and 53–120% (Fig 3C & 3D) respectively, in cardiac mitochondria of MnSOD^+/-^ (vs. WT) mice (all, ^a^p<0.05). Chronic *Tc* infection resulted in 220–250% and 35–114% increase in CI- and CII-driven DHE and amplex red oxidation, respectively, in WT.*Tc* (vs. WT) mice (all, ^b^p<0.05). An overall higher rate of mtROS production, evidenced by 50–89% increase in DHE oxidation and 30–70% increase in amplex red oxidation, was noted in chronically infected MnSOD^+/-^.*Tc* (vs. WT.*Tc*) mice (Fig 3A–3D, ^d^p<0.05). Consequently, myocardial H_2_O_2_ levels were increased by 75% in MnSOD^+/-^ (vs. WT) mice and by >200% in chagasic (vs. non-infected) WT and MnSOD^+/-^ mice (Fig 3E). Together, these results suggest that a) MnSOD deficiency and chronic *Tc* infection, individually, increase the myocardial mtROS production and ROS level, and b) MnSOD deficiency has an additive effect on *Tc*-induced mitochondrial and cardiac oxidative stress in Chagas disease.

**Fig 4:**
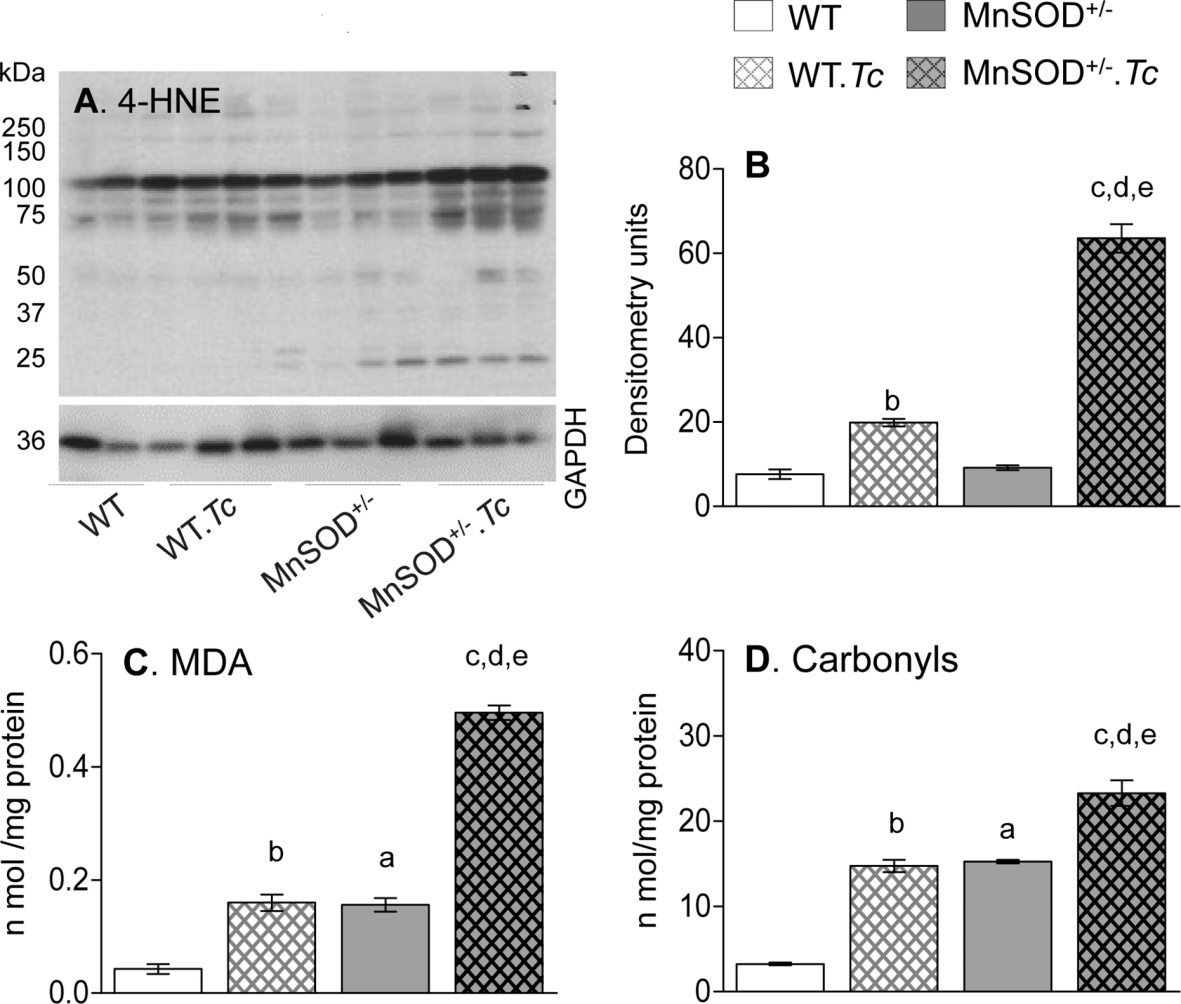
Effects of MnSOD deficiency on myocardial oxidative stress in chagasic mice. C57BL/6 (WT, MnSOD^+/-^) mice were sacrificed at 150 days post-infection corresponding to chronic disease phase. **(A&B)** Representative Western blot images for myocardial levels of 4-hydroxynonenal (4-HNE) and GAPDH (loading control) are shown (***A***, n = 3 mice per group). Densitometry analysis was performed for Western blot gels representing n = 6 mice per group (duplicate observations per mouse), and data were normalized to GAPDH levels ***(B)***. The myocardial levels of **(C)** lipid hydroperoxides / malondialdehydes (MDA) and **(D)** DNPH-derivatized carbonyl proteins were analyzed by an ELISA (n = 6 mice per group, triplicate observations per mouse). Data in bar graphs are plotted as mean value ± SEM. Significance is presented by a-e as described in Fig 1 (p value < 0.05).

We next determined the effects of increased mtROS on myocardial oxidative damage in MnSOD^+/-^ (vs. WT) mice. We examined 4-HNE that is an α, β-unsaturated hydroxyalkenal produced by lipid peroxidation, MDA that is a stable breakdown product of highly reactive lipid hydroperoxides formed by the action of ROS on polyunsaturated fatty acids, and carbonyls that are protein-derived aldehydes and ketones produced by direct oxidation of amino acids. No significant change in 4-HNE levels was noted in the myocardium of WT and MnSOD^+/-^ mice (Fig 4A), while basal levels of MDA and protein carbonyls were increased by 290–370% in MnSOD^+/-^ (vs. WT) mice (Fig 4B & 4C). In response to chronic infection, myocardial 4-HNE content was barely increased (Fig 4A), and MDA and carbonyl levels were increased by 350% (Fig 4B & 4C, ^b^p<0.05) in WT.*Tc* (vs. WT) mice. The MnSOD^+/-^.*Tc* mice exhibited maximal myocardial oxidative stress, evidenced by 230% and 57.7% higher levels of MDA and protein carbonyls, respectively, when compared to normal MnSOD^+/-^ and chronically infected WT mice (Fig 4B & 4C, ^c,d^p<0.05); and 1140% and 615% increase in MDA and protein carbonyls, respectively, when compared to normal WT mice (Fig 4B & 4C, ^e^p<0.05). The myocardial 4-HNE levels were also highest in MnSOD^+/-^.*Tc* mice as compared to any other group of mice. Together, these results suggest that MnSOD deficiency exacerbated the damaging oxidants in the myocardium of chagasic mice.

**Fig 5:**
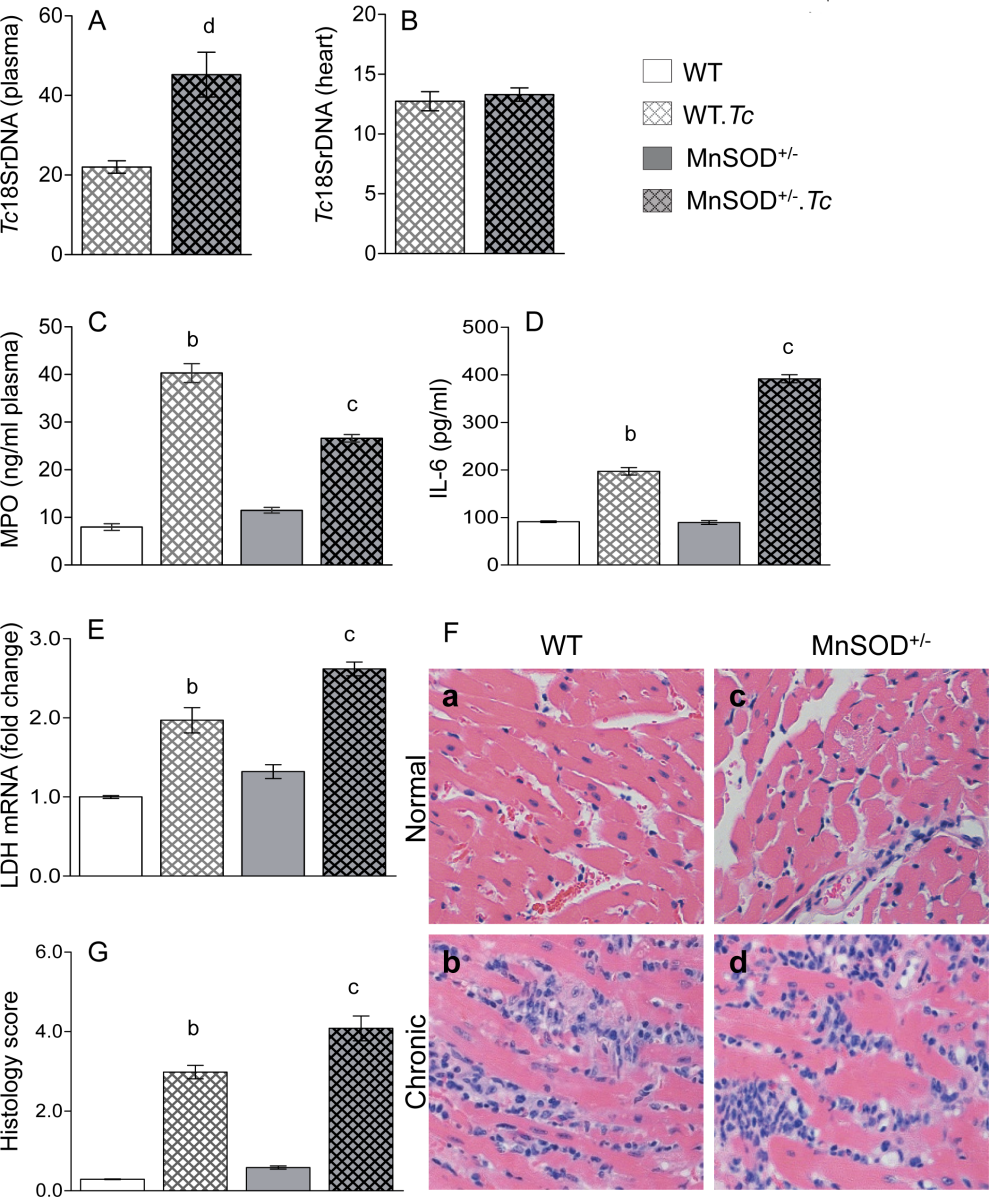
Effects of MnSOD deficiency on parasite persistence and inflammatory stress in chronic chagas disease. C57BL/6 (WT, MnSOD^+/-^) mice were sacrificed at 150 days post-infection. **(A&B)** Peripheral blood ***(A)*** and myocardial ***(B)*** total DNA was used as a template and parasite level was determined by real time qPCR evaluation of Tc18SrDNA. Data were normalized to *GAPDH*. **(C&D)** Shown are plasma levels of myeloperoxidase (MPO) activity ***(C)*** and interleukin 6 (IL-6) level in WT and MnSOD^+/-^ mice (±*Tc*). **(E)** RT-qPCR evaluation of myocardial levels of *LDH* mRNA was performed, and data were normalized to *GAPDH* mRNA. **(F)** Heart tissue-sections were subjected to H&E staining (blue: nuclear, pink: muscle/cytoplasm/keratin). Shown are representative images from non-infected (a&c) and chronically infected (b&d) WT (a&b) and MnSOD^+/-^ (c&d) mice (magnification: 20X). Histology score **(G)** was calculated as described in Materials and Methods. All data are plotted as mean value ± SEM, and acquired from 6 mice per group (triplicate observations per sample). Statistical significance is annotated as ^b^WT.*Tc* vs. WT, ^c^MnSOD^+/-^.*Tc* vs. MnSOD^+/-^ (p value < 0.05).

Finally, we examined peripheral and myocardial parasite burden, plasma levels of myeloperoxidase and IL-6, and myocardial inflammatory infiltrate and LDH expression to evaluate the effect of parasite and MnSOD deficiency on inflammatory stress in Chagas disease. These data showed higher level of blood parasitemia and equal level of myocardial parasite burden in chronically infected MnSOD^+/-^ (vs. WT) mice (Fig 5A & 5B). The plasma levels of MPO and IL-6 and myocardial expression of LDH mRNA (cellular injury marker) were increased by >300%, 90%, and 97% respectively, in chronically infected WT.*Tc* (vs. WT) mice; and by 130% 339%, and 101% respectively, in chronically infected MnSOD^+/-^.*Tc* (vs. MnSOD^+/-^) mice (Fig 5C–5E, all p<0.05). Further, histological studies showed extensive, diffused inflammatory infiltrate in the myocardium of chronically infected WT (histological score: 3.0 ± 0.41) and MnSOD^+/-^ (histological score: 4.0 ± 0.76) mice (Fig 5Fb and 5Fd). Together, these data suggest that mitochondrial deficiency of MnSOD and increased mtROS contributed to a slight increase in peripheral and myocardial inflammatory stress but it did not further exacerbate the chronic parasite persistence in the heart in Chagas disease.

## Discussion

MnSOD is a primary antioxidant located on the matrix side of inner mitochondrial membrane, wherein it dismutates the superoxide byproducts of oxidative phosphorylation (OXPHOS) to H_2_O_2_ and the latter is further reduced by glutathione peroxidases and catalase [22]. Mutations in the gene encoding MnSOD have been associated with idiopathic cardiomyopathy, aging, and cancer. In this study, we demonstrate that host deficiency of MnSOD exacerbates the mitochondrial stress in chronic chagasic cardiomyopathy of infectious etiology.

Our results in this study and other reports demonstrate that MnSOD levels were compromised with the development of chronic Chagas disease in mice [23] and humans [24]. NFE2L2 (basic leucine zipper transcription factor) binds to the antioxidant response element (ARE) in promoter region of antioxidant genes, and a decline in nuclear translocation and activity of NFE2L2 was suggested to contribute to decreased *MnSOD* expression during *T*. *cruzi* infection [14]. In another study, a therapeutic approach based on inhibition of phosphodiesterase 5 (PDE5) was shown to enhance the ROS scavenging capacity and establish antioxidant/oxidant balance in chagasic myocardium by activation of MnSOD expression and activity [25]. Further studies will be required to delineate how and if PDE5 suppresses the antioxidant capacity via targeting the NFE2L2 pathway of antioxidant response in the heart. Yet, our previous findings in WT and MnSOD^tg^ mice [12,14] and comparative analysis of WT and MnSOD^+/-^ mice in this study establish that MnSOD is essential to maintain mitochondrial respiratory function and arrest mtROS in the myocardium. We surmise that small molecule MnSOD mimetics will be beneficial in arresting the mtROS and oxidative adducts of the macromolecules such as DNA, lipids, and proteins in chagasic myocardium.

ROS contributes to hypertrophy and remodeling of the failing myocardium through multiple mechanisms. For example, ROS signals phenotypic transformation of fibroblasts to myofibroblasts and triggers fibrosis, collagenosis, and the activation of matrix metalloproteinases [26,27]. ROS-dependent formation of advanced glycation end (AGE) products accelerates the crosslinking of collagens in diabetic heart [28,29]. In other instances, ROS mediated cellular injury resulted in loss of cardiomyocytes [30]. Thus, ROS can contribute to thickening as well as thinning of the LV walls. Indeed, enhancing the MnSOD levels improved the myocardial performance index through control of ROS-induced hypertrophy and cardiac injuries in chagasic mice [14].

It is also reported that ROS, through oxidation of IκB, promotes nuclear translocation of RelA/p65 and transcriptional activation of numerous genes involved in inflammatory and proliferative responses [31]. We have shown ROS-dependent increase in the expression of inflammatory cytokines (IL-1β, TNF-α) in cardiomyocytes infected by *T*. *cruzi* [32]. A control of inflammatory responses (MPO, LDH) and myocardial inflammatory infiltrate was also observed in chronically infected MnSOD^tg^ (vs. WT) mice [12]. Our finding of a moderate increase in inflammatory stress in chagasic MnSOD^+/-^ (vs. chagasic WT) mice are in alignment with our previous findings and imply that mtROS contributes to chronic inflammatory stress in Chagas disease. It is worth noticing that sirtuin 1 (SIRT1) deficiency also predisposed the chagasic heart to chronic inflammation through increased levels of acetylated (functional) form of p65/RelA [16]. Polyadenosine ribose polymerase (PARP1) competes with SIRT1 for substrate, and an increase in PARP1-dependent cytokine gene expression was also noted in infected cardiomyocytes [33]. Consequently, treatment with PARP1 inhibitor or SIRT1 agonist arrested the NFκB-dependent inflammatory cytokine expression in infected cardiomyocytes [33] and chagasic heart [16]. We surmise that SIRT/PARP1 balance along with activators of SOD2 will provide promising new therapeutic strategies for arresting chronic oxidative and inflammatory stress and cardiac dysfunction in Chagas disease [34].

In summary, we have shown that MnSOD deficiency aggravates the mitochondrial dysfunction of electron transport chain, mtROS production, and oxidative adducts in Chagas disease. We propose that MnSOD mimetics capable of protecting the mitochondria from oxidant stress and maintain metabolic homeostasis will have potential utility as adjuvant therapy in arresting the evolution of chronic Chagas disease.

## Supporting information

S1 Table Oligonucleotides used in the study. (DOCX)
