# Supplementary material for: Manganese superoxide dismutase deficiency exacerbates the mitochondrial ROS production and oxidative damage in Chagas disease
Source: PLoS Negl Trop Dis. 2018 Jul 25;12(7):e0006687. doi: 10.1371/journal.pntd.0006687 (PMC6078326; doi:10.1371/journal.pntd.0006687)
Supplement: S1 Table — (DOCX) [file pntd.0006687.s001.docx]

**S1 Table: Oligonucleotides used in the study**

| **Gene** | **5‘-3’ Forward** | **5‘-3’ Reverse** | **Size (bp)** | **Accession #** |
| --- | --- | --- | --- | --- |
| ***RT-qPCR (mRNA)*** | |  |  |  |
| *SOD1* | CTCAGGAGAGCATTCCATCATT | CTCCCAGCATTTCCAGTCTT | 108 | NM_011434.1 |
| *SOD2* | CACATTAACGCGCAGATCATG | CCAGAGCCTCGTGGTACTTCTC | 79 | NM_013671.3 |
| *SOD3* | CTTGTTCTACGGCTTGCTACT | CAGGTCAAGCCTGTCTATCTTC | 121 | U38261.1 |
| *LDH* | GGACTTGGCTGAGAGCATAA | GATACATGGGACACTGAGGAAG | 112 | M17516.1 |
| *GAPDH* | AACTTTGGCATTGTGGAAGG | ACACATTGGGGGTAGGAACA | 223 | NM_008084.2 |
| ***qPCR (DNA)*** | |  |  |  |
| *SOD2* | TGCTGACCTGCTGGATTACA | CCTGACCAAGGAAAGCAAAG | 229 | NM_013671.3 |
| *Tc18SrDNA* | TTTTGGGCAACAGCAGGTCT | CTGCGCCTACGAGACATTCC | 197 | X53917 |
| *GAPDH* | ACAATTTCCATCCCAGACCC | CCCAACACCGCATTAAAACC | 122 | NC_000072.6 |
